# Establishment of COVID-19 testing laboratory in resource-limited settings: challenges and prospects reported from Ethiopia

**DOI:** 10.1080/16549716.2020.1841963

**Published:** 2020-11-17

**Authors:** Adugna Abera, Habtamu Belay, Aboma Zewude, Bokretsion Gidey, Desalegn Nega, Boja Dufera, Abnet Abebe, Tujuba Endriyas, Birhanu Getachew, Henok Birhanu, Hailemariam Difabachew, Bacha Mekonnen, Helina Legesse, Firdawek Bekele, Kalkidan Mekete, Seble Seifu, Heven Sime, Nebiyou Yemanebrhan, Mesfin Tefera, Hiwot Amare, Berhane Beyene, Estifanos Tsige, Adisu Kebede, Geremew Tasew, Getachew Tollera, Ebba Abate, Adugna Woyessa, Ashenafi Assefa

**Affiliations:** a Malaria and Neglected Tropical Diseases Research Team, Ethiopian Public Health Institute, Addis Ababa, Ethiopia; b National Laboratories Capacity Building Directorate, Ethiopian Public Health Institute, Addis Ababa, Ethiopia; c National Polio and Measles Laboratory, Ethiopian Public Health Institute, Addis Ababa, Ethiopia; d Influenza and Arbovirus Research Laboratory, Ethiopian Public Health Institute, Addis Ababa, Ethiopia; e Laboratory Coordinator, WHO Ethiopia Country Office, Addis Ababa, Ethiopia; f Clinical Bacteriology and Mycology Case Team, Ethiopian Public Health Institute, Addis Ababa, Ethiopia; g Ethiopian Public Health Institute, Addis Ababa, Ethiopia

**Keywords:** COVID-19, EPHI, SARS-CoV-2, Ethiopia, pandemic, laboratory, RT-PCR

## Abstract

The Coronavirus pandemic is recording unprecedented deaths worldwide. The temporal distribution and burden of the disease varies from setting to setting based on economic status, demography and geographic location. A rapid increase in the number of COVID-19 cases is being reported in Africa as of June 2020. Ethiopia reported the first COVID-19 case on 13 March 2020. Limited molecular laboratory capacity in resource constrained settings is a challenge in the diagnosis of the ever-increasing cases and the overall management of the disease. In this article, the Ethiopian Public Health Institute (EPHI) shares the experience, challenges and prospects in the rapid establishment of one of its COVID-19 testing laboratories from available resources. The first steps in establishing the COVID-19 molecular testing laboratory were i) identifying a suitable space ii) renovating it and iii) mobilizing materials including consumables, mainly from the Malaria and Neglected Tropical Diseases (NTDs) research team at the EPHI. A chain of experimental design was set up with distinct laboratories to standardize the extraction of samples, preparation of the master mix and detection. At the commencement of sample reception and testing, laboratory contamination was among the primary challenges faced. The source of the contamination was identified in the master mix room and resolved. In summary, the established COVID-19 testing lab has tested more than 40,000 samples (August 2020) and is the preferred setting for research and training. The lessons learned may benefit the further establishment of emergency testing laboratories for COVID-19 and/or other epidemic/pandemic diseases in resource-limited settings.

## Background

Coronaviruses (CoV) are a large family of viruses that cause illness ranging from the common cold to more severe diseases such as Middle East Respiratory Syndrome (MERS-CoV) and Severe Acute Respiratory Syndrome (SARS-CoV). The novel coronavirus (SARS-CoV-2) is a new strain which had not previously been identified in humans [1]. The novel coronavirus is classified as a beta-coronavirus, similar to previously discovered coronaviruses. The virus is mainly transmitted through droplets generated when an infected person coughs, sneezes, or exhales. Most people who fall sick with COVID-19 will experience mild to moderate symptoms and recover without special treatment [1,2].

The most common laboratory testing method for SARS CoV-2 is by detecting the virus nucleic material (RNA) using RT-PCR. The test uses nasopharyngeal/oropharyngeal swabs or other upper respiratory tract specimens, including sputum and saliva samples [3]. A variety of RNA gene targets are used by different manufacturers, with most tests targeting one or more of the envelopes (*env*), nucleocapsid (*N*), spike (*S*), RNA-dependent RNA polymerase (*RdRp*), and *ORF1* genes. The sensitivities of the tests to individual genes are comparable [4].

As of 12 July 2020, the COVID-19 pandemic has affected more than 12.9 million people worldwide resulting in more than 567,914 deaths [5]. In Ethiopia, the disease is spreading at an alarming rate. The first case was reported on 13 March 2020, since then 250,604 people have been tested, 7,402 people have been confirmed by laboratory tests to be infected by SARS-CoV-2 and 124 lives have been lost. The number of infected cases reported is increasing daily [6].

## Limited national testing capacity and call for expansion

When COVID-19 was spreading out of Wuhan, China in January 2020, Ethiopia had limited capacity in the molecular diagnosis of coronaviruses. With unprecedented effort and as the lead national referral laboratory, the EPHI started managing testing for COVID-19 by early March 2020. However, the limited testing capacity at EPHI could not keep pace with the increasing demand for testing. Initially, a COVID-19 testing laboratory was initiated by the Virology/Influenza team at the EPHI. Following the quest for expansion of testing at the EPHI, the Malaria and Neglected Tropical diseases (NTDs) research team at EPHI also took the initiative to establish a complementary COVID-19 testing laboratory with the available resources initially allotted for Malaria and NTD research activities.

In addition, several public universities, research institutes and hospitals in the country joined the effort by establishing their own COVID-19 testing laboratories. Herein, we have documented the processes followed and challenges encountered during the successful establishment of the COVID-19 laboratory by the Malaria and NTDs research team at the EPHI.

## Capacity of the Malaria and NTDs laboratory in terms of molecular biology

The Malaria and NTDs team at EPHI had limited capacity in molecular testing. The existing resource focus was on Chelate extraction capacity and molecular testing of drug resistance using conventional thermocycler and gel electrophoresis. The laboratory was mainly inactive due to weak procurement systems for molecular reagents. However, the key equipment for molecular work including thermocycler, centrifuges, vortex, hotplate and pipettes was available in the laboratory.

## Approach

To develop clear structures of accountability and leadership, a steering committee was formed by voluntary team members from the Malaria and NTDs research team to lead the laboratory establishment. The nucleus team comprised of the laboratory manager, quality officer, safety officer and the team leader. The group started identifying space in parallel with mobilizing materials and consumables. Available equipment and consumables in the Malaria and NTD research laboratory and additional consumables provided by the national Emergency Operation Centre (EOC) targeting COVID-19 testing were taped and utilized for establishment.

## Selection and renovation of standard rooms

COVID-19 molecular laboratory testing has its own set of safety requirements with the need to have at least level two safety cabinets or equivalents [5]. The standard extraction and amplification laboratory rooms used by the Malaria and NTDs team are amid offices and are not convenient for handling contagious samples similar to COVID-19. As a result of the inadequacy of the existing laboratory space for contagious material handling, a search and identification process for appropriate rooms that meet the required safety standards was initiated. Consequently, a space previously used as a washing room for the Ethiopian National AIDS Research Program (ENARP) but in current marginal use was identified at the back of the Malaria and NTDs laboratory. The Malaria and NTDs team was planning to renovate the washing room for an NTDs molecular study. The team of experts found the site appropriate for contagious materials management and received permission from the institute’s authorities together with strong backing from the top management. The washroom mentioned above was a large room with three access doors. The team partitioned the room into five dedicated rooms for sample reception, RNA extraction, master mixing, detection and amplification, and data management. To ensure safety and security, the entry corridor was outfitted with automated lockable doors to restrict the movement of unauthorized persons. World Health Organization (WHO) documents and experiences from the virology units at the EPHI were consulted to design the room’s structure and workflow. The rooms were partitioned with aluminium metal frames and glass windows with adjoining entrance doors positioned in the middle of each room. Two 4 m × 4 m rooms and three smaller 2 m × 2 m rooms were fully setup and painted within two weeks.

**Figure 1.**
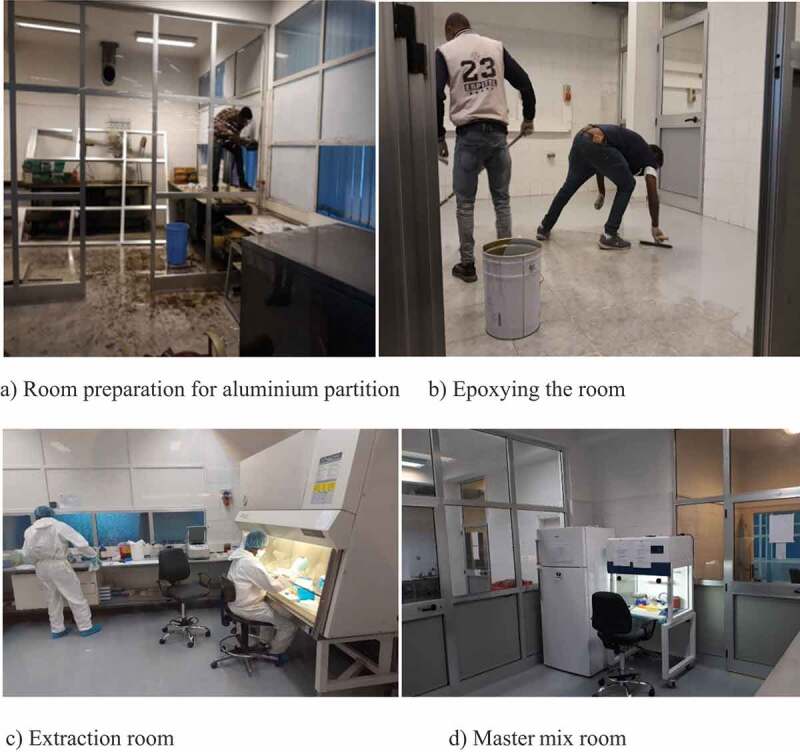
Parasitology COVID-19 testing laboratory. (a) Room initial partitioning with aluminium and glass. (b) Room floor painting with epoxy for smoothening the floor for better cleaning. (c and d) extraction and master mix room in use

## Infrastructure and equipment

Simultaneously with room renovation, the team started locating and collecting standard materials required for the new laboratory. The EPHI is a historic institute (more than 100 years since establishment) and large institute with significant human potential and equipment [6]. The first two resources located within the institute were i) a level II biosafety cabinet at the malaria culture laboratory and ii) an ABI 7500 Real-time PCR machine owned by the entomology team, both were rarely used and the latter demonstrated some defects during testing. The cabinet and PCR machine were moved to the renovated COVID-19 laboratory space. The cabinet was recalibrated and tested by biomedical engineers to control the effect of movement on the standard functioning of the instrument. Experts from the Polio and Influenza teams at EPHI helped with calibrating the RT PCR machine. During the first trial test, the ABI 7500 RT PCR was malfunctioning. As a result, a concerted effort was made to solve the issues at hand by communicating with local as well as international colleagues and the ABI bio-system agents. After an intensive period of problem identification and diagnosis by the team, it was concluded that the machine had an electric system problem and required calibration. The team fixed the electric system problem and searched for calibration kits for the ABI 7500 biosystem RT PCR machine. There was a keen awareness that the new procurement of kits may require ample budget and considerable time with the current COVID-19 global slowdown. Fortunately, one of the experts had the required reagents left over from recent calibration and hence the RT PCR machine got calibrated. The machine was tested with plates prepared in another EPHI laboratory (Influenza) with known samples and proved to be working. However, on the second day of the machine calibration, a power fluctuation damaged the desktop computer with the ABI 7500 biosystem software and the window failed. Providentially, the RT PCR machine was not plugged at the time of fluctuation and survived the accident. The nucleus team installed accessible window and ABI 7500 biosystem software. After several trials and examinations, the ABI 7500 RT PCR machine was recalibrated and passed the major requirements for testing. Around the same time, a new QuantStudio 5 RT PCR which was previously acquired for a research project on schistosomiasis arrived for the team’s use. The QuantStudio 5 RT PCR machine’s manual reading showed that the machine was partially calibrated, and the required program could be downloaded online. The QuantStudio 5 instrument was ready to use with minimal calibration and the required software was installed safely.

In parallel, part of the team was mobilizing molecular laboratory instruments such as pipettes, tips, vortex, centrifuges, spines, hotplates, biohazard bags and other consumables to start the actual testing. Most of them were collected from the Malaria and NTDs laboratories at EPHI.

In the process of infrastructure and equipment organization, the team benefited from and was capacitated in leadership, team work, critical thinking in problem solving and leveraging human and material resources at the institute.

## Organizing the lab

The establishment team generally agreed to have a separate setup for dirty rooms (sample reception and extraction) and clean rooms (master mix). Sample flow was organized in such a way that the dirty rooms do not contaminate the clean rooms. Maximum efforts were exerted to prevent contamination and infection from the dirty rooms (Figure 1).

**Figure 2.**
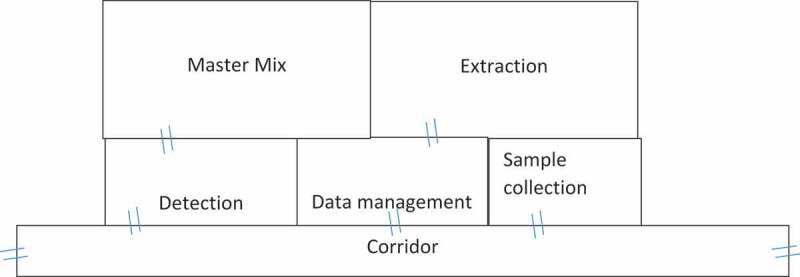
Parasitology COVID-19 testing laboratory floor design (The double lines are indicative of doors)

## Consumables and reagents

PCR reagents and Personal Protection Equipment (PPE) were made available through the national COVID-19 response team and aid from the Chinese philanthropist Jack Ma [7,8]. The team had received training and support from available virology experts where junior and senior staff were trained and capacitated in molecular biology skills. When the laboratory and personnel were ready, the team decided to receive samples. Readiness was declared and the first 20 samples received were collected from community surveys.

The collected samples were extracted using Da An nucleic acid extraction kit (Da An Gene Co., Lt. of Sun Yat-sen University); a master mix was prepared using Da An detection kit for Novel Coronavirus (2019-nCoV) RNA (Da An Gene Co., Lt. of Sun Yat-sen University) and amplified. The results were twofold; the system worked but the result was undetermined and non-interpretable. Similar tests and results were repeated two to three times. Potential contamination from unknown source was suspected. In the meantime, since the setup of the laboratory was convenient, experts from EPHI’s virology unit requested to extract their samples using the extraction facility, this was in part to avoid the overcrowding at the virology laboratory. The extracts were all contaminated when tested in the newly established laboratory. The nucleus team started searching for the source of the contamination one by one.

## Extraction room

The entire extraction room, the safety cabinet and all materials including freezers were cleaned using bleach and 70% alcohol to control contamination from nucleic acid and other cellular contaminants that may interfere with assay detection. The room was fumigated with formaldehyde and neutralized. The safety cabinet was exposed to UV light and the laminar fan was kept turned on and running for a couple of days.

## Amplification and detection rooms

In the amplification room, all the pipettes were calibrated per the national standards and exposed to UV light; the door to the extraction room was closed and human movement was restricted, except for authorized persons. All the rooms were thoroughly cleaned and fumigated.

## Major challenges faced

The team ran the experiment again; however, the result was further frustrating as it showed that the contamination had not yet been removed. The situation was considered as a total laboratory contamination and a corrective action plan was agreed upon. Given the inability to turnaround the samples in the expected timeframe, the COVID-19 national coordinating team notified the Malaria and NDTs team to return the samples. Repeated attempts at extraction and detection failed following which the samples were returned by keeping aliquots. The search for the source of contamination continued. At this stage, most colleagues were frustrated and little to no support remained.

To mitigate the challenges, an experiment was designed to extract the samples and master mix in different COVID-19 testing laboratories at the EPHI and the detection was run using different instruments (Table 1).

**Table 1.** Experimental setup to mitigate laboratory contamination in two laboratories

	Extraction	Master mix	Detection	Result
Experiment 1	Lab A	Lab A	Lab A	Failed
Experiment 2	Lab B	Lab A	Lab A	Failed
Experiment 3	Lab A	Lab B	Lab A	Succeeded
Experiment 4	Lab A	Lab B	Lab B	Succeeded
Experiment 5	Lab B	Lab B	Lab A	Succeeded

Lab A is the parasitology COVID19 testing laboratory. Lab B is the clinical bacteriology and mycology reference laboratory.

The results indicated that the source of the contamination was the master mix room and potentially the master mix cabinet, particularly the ill working internal airflow. The team decided to remove the safety cabinet from the laboratory and kept on searching for laminar flow from different laboratories. At the same time, a master mix was prepared without the cabinet and limited contamination was found. Whereas samples were requested again for processing by the Malaria and NTDs team, the national COVID-19 testing team was hesitant to accommodate the request. The team received a few samples, ran experiments, and observed encouraging results. Before releasing the results, the team found a new laminar flow fitted with a UV light from one of the sister laboratories at EPHI. The experiment was repeated, and the results were confirmed. The results were confirmed in another laboratory and finally the new laboratory under the Malaria and NTDs team joined the chain of national COVID-19 testing laboratories with an initial testing capacity of 50–100 samples per day, with turnaround time of 24 hours. The new laboratory adopted a sample pooling strategy with four samples pooled together and only pools that have tested positive are individually extracted while the negative pools being reported as negative samples [9]. The establishment is working on the expansion of human resources and providing training to other similar laboratories from different regions including universities and hospitals.

## Current status

The laboratory design was revised (Figure 2), it is receiving continuous supply of samples from the national COVID-19 testing unit and is working in three shifts reaching a testing capacity of up to 700 samples per day. The new laboratory is now part of the national COVID-19 testing chain and has become a preferred training and research unit because of its convenient setup and 20 skilled permanent and temporary staff. Thus far (31 August 2020), the laboratory has tested more than 39,326 samples total, including more than three thousand positive samples. It has also trained 30 individuals from different universities and hospitals in the country. The national testing capacity has reached around 20,000 per day and a total of 1.1 million samples have been tested nationally. A chain of 54 laboratories that include research institutes, universities, hospitals, and regional health offices have already been established all over the country. During the current national testing campaign, Community-Based Activity and Testing (ComBAT), the EPHI-Parasitology (PCL) laboratory was recognized as the best performer amongst COVID-19 testing laboratories in the country (see additional supplement document). COVID-19 is expected to wane over time, research efforts are being developed to use the capacity built towards research in COVID-19 and parasitic diseases co-infections. Regarding COVID-19 research, a proposal for a sample pooling validation study that was submitted by the Malaria and NTDs team was recently approved by the EPHI-IRB. Additionally, a study proposal has been submitted for serology/ELISA-based COVID-19 testing and research is ongoing to compare and validate four reagents to provide input for informed decision-making.

## Laboratory linkage

**Figure 3.**
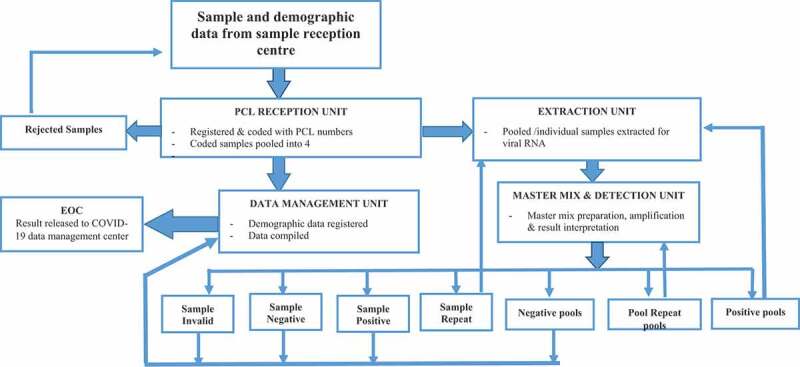
Parasitology COVID-19 testing laboratory design and workflow (PCL: Parasitology COVID Laboratory; EOC: Emergency Operation Centre): Naso/Oro-pharyngeal samples collected by trained health care professionals from risk groups, healthcare facilities, airports, border areas and communities are transported in standard triple package via cold chain to the sample reception centre. Samples allocated to PCL are received using standardized sample receiving/rejection format. Rejected samples are communicated to sample reception centre. Standard samples are given laboratory IDs and transferred to the Extraction unit. Extracted samples then transferred to master mix and detection unit where samples loaded with reagent to be detected by RT PCR machine. Results communicated to EOC through data management unit

The Emergency Operation Centre (EOC) of the centre for Public Health Emergency Management (cPHEM) at EPHI is the coordinating body for COVID-19 surveillance, diagnosis, and response. The laboratory section at the EOC coordinates the sample collection from health facilities, communities, congregate settings and vulnerable groups. The sample management unit at the EOC receives samples collected by trained health professionals using standard viral transporting medium (VTM) reinforced by triple packaging, following receipt the samples get registered and distributed to COVID-19 testing laboratories in Addis Ababa. After sample processing, using the Demographic and Health Information System-2 (DHIS-2) platform negative results dispatched to patients via short message service (SMS) and by email to institutions whereas the case management unit of the EOC communicate positive results to the relevant entities for appropriate action (Figure 3).

## Lessons learned

The new laboratory is named Parasitology Covid-19 testing Laboratory at EPHI (PCL-EPHI). With a strong commitment, teamwork, leadership and strong management support, an effective COVID-19 testing laboratory was established in a resource-limited setting, at a point in time when laboratory capacity expansion was most critical. Some of the lessons learned include: 1) Repurpose existing laboratory spaces using national and WHO guidelines 2) Mobilize underutilized resources such as equipment and human resources for COVID-19 laboratory setup. Particularly equipment available for health research and diagnostics 3) Collaborate with local and international health experts and equipment manufacturers and agents to solve laboratory issues 4) Ensure that space and equipment identified for COVID-19 testing is sterile and contamination free from the outset. Universities (there are 40 public universities in Ethiopia), hospitals and research institutes may follow a similar approach to establish COVID-19 testing and/or other emergency laboratories using available resources.

## Conclusion

Currently, COVID-19 is increasingly spreading in Ethiopia as part of the global pandemic. The Ethiopian population is estimated to be 114 million [10] and the government is calling for further testing COVID-19 capacity. The lessons learned in this case can be used to further establish COVID-19 testing laboratories in Ethiopia and in other resource-limited settings. On the other hand, using the opportunity the current pandemic has created, the EPHI benefited from the establishment of an additional standard molecular laboratory, capacitated staff as well as the identification of its human and material resources that may last beyond the COVID-19 era.

## Paper context

Limited molecular laboratory capacity in resource constrained settings is a challenge in the diagnosis of the ever-increasing cases and the overall management of COVID-19. This paper explores the experience, challenges and lessons learned in renovation and establishment of a capacitated rapid molecular testing laboratory for the diagnosis of SARS-CoV-2 in a resource limited setting.
